# Additional Rehabilitative Robot-Assisted Gait Training for Ambulation in Geriatric Individuals with Guillain–Barré Syndrome: A Case Report

**DOI:** 10.3390/medicina60081209

**Published:** 2024-07-26

**Authors:** Fred Yi-Shueh Chen, Wen-Hsuan Hou, Hsun-Hua Lee, Ying-Chi Huang, Co Yih Siow

**Affiliations:** 1 Department of Physical Medicine and Rehabilitation, Taipei Medical University Hospital, Taipei 11031, Taiwan; 2 Department of Physical Medicine and Rehabilitation, School of Medicine, College of Medicine, Taipei Medical University, Taipei 11031, Taiwan; 3 Cochrane Taiwan, Taipei Medical University, Taipei 11031, Taiwan; 4 Department of Geriatric Medicine, Taipei Medical University Hospital, Taipei 11031, Taiwan; 5 Department of Neurology, Taipei Medical University Hospital, Taipei Medical University, Taipei 11031, Taiwan; 6 Department of Neurology, School of Medicine, College of Medicine, Taipei Medical University, Taipei 11031, Taiwan; 7 Department of Neurology, Vertigo and Balance Impairment Center, Shuang Ho Hospital, Taipei Medical University, New Taipei City 23561, Taiwan

**Keywords:** Guillain–Barré syndrome, geriatric, robot-assisted gait training, case report

## Abstract

We present a case of a 75-year-old Asian woman with Guillain–Barré syndrome (GBS) who underwent a 1-month comprehensive rehabilitation training program supplemented by robot-assisted gait training (RAGT). GBS can lead to fatigue and prolonged bed rest, thereby further debilitating older patients. Although exercise intervention is recommended for GBS, a consensus regarding the appropriate intensity has yet to be established. Individualized strategies are required because older patients experience varying levels of fatigue and frailty. We used a technological adjunct to support comprehensive rehabilitation for GBS reconditioning in an older patient. To the best of our knowledge, research involving the use of an exoskeleton robotic device in the geriatric population with GBS is limited. Our case demonstrates the feasibility and safety of RAGT for improving lower limb muscle power and scores on the Barthel Index, Clinical Frailty Scale, and Instrumental Activities of Daily Living Scale at discharge from a geriatric ward.

## 1. Introduction

Guillain–Barré syndrome (GBS) is an acquired immune-mediated inflammatory disease of the peripheral nervous system, and it has been reported to have an annual global incidence of approximately 1–2 cases per 100,000 person-years [1,2]. A predictive factor for poor outcomes in GBS is older age [3,4]. The incidence of GBS increases with age, and the recovery period is longer for older patients than for young adults [2,5]. Therefore, managing older patients with GBS can be highly challenging.

GBS can cause flaccid paralysis, resulting in difficulties with talking, swallowing, breathing, and walking. Even with appropriate pharmacological treatment, approximately 10–20% of patients experience residual disabilities because of muscle weakness, and approximately 60–80% experience fatigue after GBS [6,7,8]. Because GBS can be debilitating, rehabilitation is required for recovery to reduce the risk of disability, restore physical function, and improve patients’ ability to engage in activities of daily living (ADLs).

A consensus has yet to be reached regarding the exercise protocol for patients with both GBS and disabilities. One study suggested that high-intensity exercise is more effective than low-intensity exercise in reducing the occurrence of disability [9]. However, exercise must be implemented with caution because overtraining denervated muscles in patients with peripheral nerve involvement may cause further weakening and fatigue [10,11,12].

Herein, we report a case of an older patient with GBS who was admitted to the geriatric ward of a university hospital for multidisciplinary care. Comprehensive geriatric assessment (CGA) was conducted to evaluate functional and cognitive abilities and to identify potential disabling conditions affecting physical and mental health [13]. Rehabilitation was implemented to reduce functional deficits, impairments, and disabilities. Performing gait training with conventional rehabilitation is technically difficult because of motor weakness and balance problems associated with the flaccid paralysis caused by GBS. Therefore, to increase the intensity of our patient’s treatment, a robotic-assisted gait training (RAGT) device Lokomat (Hocoma AG, Volketswil, Switzerland) was incorporated into our rehabilitation program. This is a rare case of an older patient undergoing technological therapy adjunct with conventional rehabilitation during the process of recovering from GBS. Our objective is to present the feasibility of additional RAGT for ambulation and evaluate its improvement in the CGA of an elderly GBS patient. The case report has been reported in line with the CARE guidelines [14].

## 2. Patient Information

A 75-year-old Asian woman was admitted to a university hospital for treatment of GBS in January 2024. She initially presented with symmetric weakness in both distal lower limbs and ataxia. Physical examination of deep-tendon reflexes in the knee and ankle showed areflexia. She also reported experiencing numbness and a burning sensation in the bilateral distal lower limbs. The weakness and numbness progressed quickly, ascending to the bilateral proximal thighs within a few days, and consequently she could not stand. Her upper limbs had an intact muscle power of 5, but her bilateral lower limbs had a decreased muscle power of 0 on manual muscle testing. In addition, she experienced constipation and progressive urine retention. There was no facial or other cranial nerve involvement. Magnetic resonance imaging was performed to exclude localized spinal cord lesion or cauda equina syndrome. A review of her occupational and medical history revealed that she was a housewife who had recently contracted coronavirus disease 2019, which caused her to experience mild fever and upper respiratory infection symptoms such as cough and rhinorrhea. After she was assessed by a neurologist, a nerve conduction study (NCS) was ordered. The results revealed an absence of motor conduction velocity and amplitude over the left peroneal nerve and decreased values on the right side. F-wave reflexes were absent in the bilateral peroneal nerves and prolonged in the bilateral tibial nerves. H-reflex latency was absent in the left lower limb and prolonged in the right lower limb. In addition, an elevated cerebrospinal fluid protein level was detected, and so the diagnosis of an acute inflammatory demyelinating polyradiculoneuropathy variant of GBS was confirmed.

She was treated with intravenous immunoglobulin (IVIG) at a dose of 0.4 g per kilogram of body weight per day for 5 days. Because of the paralysis in the patient’s lower limbs and her inability to adequately perform ADLs, a therapeutic program supported by a multidisciplinary team comprising a rehabilitation physician, geriatrician, physiotherapist, and occupational therapist was implemented to facilitate her care. Initial CGA showed mild depression, severe frailty, total dependency, risk of fall, malnutrition, sarcopenia, and decreased functional abilities mainly attributed to her lower limb weakness (Table 1). She underwent a comprehensive patient-centered rehabilitation program that included physical therapy and occupational therapy to improve her lower limb muscle strength, sitting balance, transfer technique, standing balance, ambulation, and gait balance. Bedside training was conducted from Monday to Friday, with a gradual increase in duration to a total of one hour, and was adjusted accordingly on the basis of her physical condition during each session. Subsequently, the patient achieved the initial goal of the program of being able to roll over and sit up on the bedside with moderate assistance from caregiver 1 week after admission. Her rehabilitation moved to a physiotherapy and occupational therapy room in the following weeks. A tilting table was used to facilitate weight bearing, and arm cycling was implemented as low-level aerobic exercise to enhance her physical condition. A Foley catheter for her urinary retention was successfully removed 2 weeks after admission and by this time her lower limb muscle power had gradually improved to 2.

**Figure 1:**
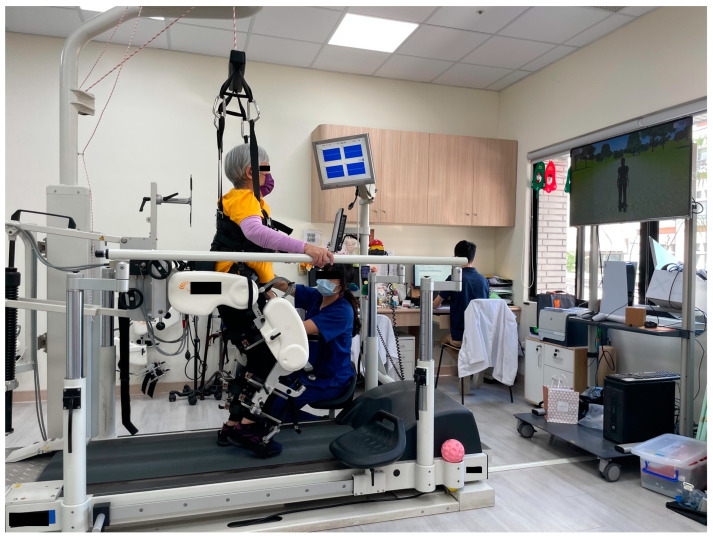
Robotic-assisted gait training (RAGT) device Lokomat. The patient is loaded onto Lokomat with the assistance of a trained physiotherapist. Augmented feedback with walking scenario provides increased attention and participation of the patient.

To further improve exercise intensity and lower limb function for the patient’s ambulation and gait balance, the RAGT device Lokomat was incorporated into her conventional rehabilitation therapy. With the help of the output from the robotic exoskeleton, the patient was able to attempt treadmill walking before she recovered her full lower limb muscle power and physical strength. Figure 1 demonstrates the patient on the treadmill using the RAGT device Lokomat. The patient is loaded onto the device and parameters including the speed and the amount of guidance force and body weight support are adjusted by a trained therapist. Guidance force determines the extent to which the patient’s movements are guided by the Lokomat orthoses while walking. Body weight support decreases the amount the patient needs to support with their legs, which is beneficial for subjects with lower trunk weakness and difficulty in maintaining an upright position. During RAGT, the therapist begins by making sure that the patient’s gait is safe, performing adjustments to parameters, and setting the gait goal for the therapy session.

Our patient underwent a total of two RAGT sessions using Lokomat, once each in the third and fourth week of admission. Prior to starting RAGT, she had already demonstrated stable vital signs during conventional rehabilitation on a tilt table for weight bearing and low-level aerobic exercises. RAGT was implemented as additional rehabilitation therapy to increase exercise intensity and begin ambulation movement. To avoid training the patient to exhaustion, RAGT was started when she was in a nonfatigued condition and terminated whenever dyspnea, fatigue, or unstable vital signs were noticed. Fatigue was assessed through patient response, and it was determined by the therapist if increased assisting guidance force or body weight support was needed or poor gait pattern developed. As determined on the basis of her ability, she initially received both 80% body weight support and guidance force, with the walking speed set at 1.2 km/h during RAGT. At this setting, she could walk a distance of 421 m in 24 min, with two breaks in between due to patient expression of fatigue. She was able to continue training after 2 min of rest each time. Her first therapy session required becoming used to the Lokomat orthoses and the feeling of relearning to walk, which involved contracting and activating muscles for specific ambulation movements. The third CGA was performed after her first RAGT attempt. Her bilateral lower limb muscle power increased to 3, and there was improvement in her Barthel Index (BI) score, from total to severe dependency. Her second RAGT session was performed in the fourth week of admission. Further improvement was achieved in her second session, during which her body weight support was decreased to 70% and her walking speed increased to 1.3 km/h. Her walking distance improved to 623 m, with an extended training duration of 30 min and no rest required. Adherence and tolerability were assessed based on patient feedback, and she was monitored for prolonged rest periods, lack of participation, or refusal to continue. She adhered to the daily conventional rehabilitation program, and there were no adverse events when an additional RAGT session was included on the same day. Whenever she experienced fatigue, the training would be paused until she felt ready to continue. Training to over-exhaustion was avoided at all times.

After 1 month of hospital admission, she improved from being bedridden to regaining the ability to sit and was able to transfer out of bed to upright standing with walker support. Her lower limb muscle power increased from 0 to 4. Follow-up outcome of the CGA at the time of discharge revealed an improvement in her BI score from 15 to 65 (listed in Table 1), indicating moderate dependency. To walk short distances, she required rollators and moderate assistance from a caregiver.

## 3. Discussion

Clinical manifestations of GBS can range from mild muscle weakness to complete muscle paralysis, which may result in impaired walking ability and cause functional deficits [26]. Advanced age is an independent risk factor related to worse prognosis and additional geriatric syndromes that can increase the risk of adverse events [5]. The European Academy of Neurology and the Peripheral Nerve Society combined task force recognizes the importance of physiotherapy, occupational therapy, speech therapy, and rehabilitation treatment during the acute and chronic phases of GBS [27]. Exercise improves the physical fitness of patients with GBS, which can enhance their physical outcomes (e.g., functional mobility) and reduce their fatigue [9,12,28,29,30]. However, few studies have explored increasing exercise intensity with RAGT for older patients with GBS. This is the first case report of RAGT being safely incorporated into the rehabilitation program of an older patient.

Acute onset rehabilitation is focused on preventing complications of immobilization, such as contractures and pressure sores, and facilitating pain management. Training includes transfer and upright posturing exercises, respiratory function training, gentle stretching, and joint mobility training. When a patient’s ability to bear weight gradually improves, they are encouraged to perform low-level aerobic exercises and undergo resistance training. The general goal of rehabilitation for patients with GBS is early mobilization without exercising to fatigue, which can help them reduce the progression of disuse atrophy and other complications related to prolonged bed rest [10,12]. Only a few studies have investigated applying RAGT for treating peripheral nerve injury caused by GBS. A literature review revealed that Lokomat-supported RAGT was successfully integrated into rehabilitation programs for two children with GBS [31,32]. Another study investigated the use of a different robotic exoskeleton on two adults with GBS and demonstrated that wearable devices are safe for adults with this condition [33]. A clinical trial was conducted to determine the effects of RAGT on nonambulatory patients with GBS who are younger than 65 years, with results still pending [34]. Our case revealed that rehabilitation supplemented by RAGT can improve the ADL performance and physical fitness of older patients recovering from GBS.

CGA results have the benefit of identifying geriatric conditions and can be coupled with tailored interventions such as rehabilitation, education, counseling, and supportive services. Our patient had intact cognitive function represented by CAM and MMSE; thus, her rehabilitation was focused on improving lower limb function, reconditioning, and preventing complications related to reduced mobility. The improvement of lower limb function is a slow process. The addition of RAGT in our case is a form of rehabilitation that is in accordance with the rationale for increased exercise intensity [9]. RAGT provides the benefit of early rehabilitation and the ability to perform gait motion even before the subject has gained lower limb muscle power for standing. Muscle activation, assisted movement, and contraction exercises through both conventional rehabilitation and RAGT are important for improving the muscle power needed for ambulation. Our patient’s improved lower limb muscle power was reflected in the CGA components related to ambulation, balance, and physical function (Table 1). Enhanced CGA is related to reduced mortality and decreased use of nursing homes and acute care hospitals [13]. Our patient achieved increased BI, IADL, SPPB and reduced SACR-F, GDS, CFS scores, all of which are positive indicators for better functional and mental health (Table 1). At discharge, she was able to transfer out of bed, which considerably reduced her caregiver burden and facilitated her participation in activities. This is consistent with the systematic review on the efficacy of rehabilitation in people with GBS, which concludes that rehabilitation improves patients’ well-being [30].

The patient was satisfied with both RAGT sessions. In her first attempt, she felt the body strap providing body weight support was uncomfortable at times. This may have caused her increased feeling of fatigue and her request to pause for rest and adjustment. However, after the first session, she was motivated because the body weight support and guidance force allowed her to be upright and gave her better foot–ground clearance. She was able to simulate walking, which she could not perform with conventional rehabilitation exercise due to insufficient lower limb muscle power. The opportunity to walk motivated her and improved her mood. In addition, the television monitor in front (as depicted in Figure 1) provided augmented feedback and walking simulation exercises, which increased her attention and participation. She was excited for the second session and experienced positive results, including improved distance, speed, and endurance. According to her experience, RAGT provided a change to her regular conventional training and was personalized to her physical needs.

The strength of this case report is that this is the first presentation of RAGT in an elderly GBS patient with positive patient feedback. RAGT allowed our patient to relearn to walk, which improved her depressive mood and increased her ability to perform movements beyond the capabilities of her earlier deconditioned status. However, a limitation in this case was the low number of RAGT intervention sessions that were used during the acute phase of GBS. Nevertheless, in the current case, a robotic-assisted device was helpful because it provided output force for limb movement even before the patient achieved adequate motor control. We adhered to a two-phase rehabilitation process, where the first phase prioritized reducing the disability burden and the second phase focused on reconditioning [9,12]. To avoid training the patient to the point of fatigue, RAGT was used in the later stage of rehabilitation, which limited the number of intervention sessions that could be conducted. When the patient regained sufficient active movement, the focus of her therapy shifted to improving her ability to perform ADLs. The number of RAGT sessions may be further increased on the basis of the patient’s condition as she recovers. RAGT effectiveness in GBS requires more study and is currently not a replacement for conventional rehabilitation.

## 4. Conclusions

We demonstrated the feasibility of integrating RAGT with conventional rehabilitation for older patients with GBS, without adverse events and with better functional outcome at discharge. Exercise rehabilitation and RAGT led to improved lower limb muscle power, which translated to better ambulation and increased independence in performing ADLs as indicated by enhanced CGA results. To increase the intensity of training through the incorporation of RAGT, a patient-centered program should be developed, since older patients may experience varying levels of post-GBS fatigue and frailty. We recommend a comprehensive assessment for the geriatric population with consideration of additional RAGT when the initial goals of bedside transfer and adequate sitting balance are achieved. Clinicians may consider applying RAGT for older patients with GBS; however, caution should be exercised to avoid fatigue or overexertion during training. Further trials should be conducted to determine the optimal dose, frequency, intensity, and efficacy of RAGT in GBS.

## Figures and Tables

**Table 1:** Comprehensive geriatric assessment scores of the patient throughout onset of Guillain–Barré syndrome.

	Baseline (Prior to Admission)	2 January 2024	19 January 2024	30 January 2024 (Discharge)
Confusion Assessment Method (CAM) [15]	0	0	0	0
Geriatric Depression Scale (GDS) [16]	1	5	5	1
Clinical Frailty Scale (CFS) [17]	3	7	7	6
Morse Fall Scale (MFS) [18]	0	45	45	60
MNA-SF * [19]	14	10	10	11
SARC-F * [20]	0	8	8	7
Barthel Index (BI) [21]	100	15	35	65
IADL * [22]	8	1	2	4
Braden Scale [23]	23	21	19	21
MMSE * [24]	30	29	29	30
SPPB * [25]	12	0	0	2

* MNA-SF, Mini Nutritional Assessment Short Form; SARC-F, Strength, Assistance in Walking, Rise from a Chair, Climb Stairs, and Falls questionnaire; IADL, Instrumental Activities Of Daily Living Scale; MMSE, Mini Mental State Examination; SPPB, Short Physical Performance Battery.

## Data Availability

The data presented in this study are available upon request from the corresponding author.
